# Supplementary material for: Prevalence of zolpidem use in France halved after secure prescription pads implementation in 2017: A SNDS database nested cohort study
Source: PLoS One. 2020 Feb 19;15(2):e0228495. doi: 10.1371/journal.pone.0228495 (PMC7029860; doi:10.1371/journal.pone.0228495)
Supplement: S1 Table — (DOC) [file pone.0228495.s001.doc]

**Supporting information**

**S1 Table. Mc Nemar test regarding changes in prevalence consumptions (sensitivity analysis).**

| Consumer in period 1 | Consumer in period 2 | | |
| --- | --- | --- | --- |
|  | No | Yes | Total |
| No | 548,186 (96.93%) | 2,161 (0.38%) | 550,347 (97.31%) |
| Yes | 9,218 (1.63%) | 6,004 (1.06%) | 15,222 (2.69%) |
| Total | 557,404 (98.56%) | 8,165 (1.44%) | 565,569 (100.00%) |

Mc Nemar’s Test : Chi-Square 4376.6, DF 1, p-value <0.0001
